# Somatic Mutation Profiling of Papillary Thyroid Carcinomas by Whole-exome Sequencing and Its Relationship with Clinical Characteristics

**DOI:** 10.7150/ijms.50916

**Published:** 2021-04-26

**Authors:** Tingyue Qi, Xin Rong, Qingling Feng, Hongguang Sun, Haiyan Cao, Yan Yang, Hao Feng, Linhai Zhu, Lei Wang, Qiu Du

**Affiliations:** 1 Department of Ultrasound, Medical Imaging Center, the Affiliated Hospital of Yangzhou University, Yangzhou University, Yangzhou 225012, China.; 2 Department of Critical Care Medicine, the Affiliated Hospital of Yangzhou University, Yangzhou University, Yangzhou 225012, China.; 3 Department of Thyroid and Breast Surgery, the Affiliated Hospital of Yangzhou University, Yangzhou University, Yangzhou 225012, China.; 4 Department of Pathology, the Affiliated Hospital of Yangzhou University, Yangzhou University, Yangzhou 225012, China.; 5 Department of Neurosurgery, the Affiliated Hospital of Yangzhou University, Yangzhou University, Yangzhou 225012, China.; 6 Central Laboratory, the Affiliated Hospital of Yangzhou University, Yangzhou 225012, Yangzhou University, China.

**Keywords:** Papillary thyroid carcinomas, Whole-exome sequencing, Genetic mutations, Cluster analysis, Ultrasonography.

## Abstract

The incidence of papillary thyroid carcinomas (PTCs) has increased rapidly during the past several decades. Until now, the mechanisms underlying the tumorigenesis of PTCs have remained largely unknown. Next-generation-sequencing (NGS) provides new ways to investigate the molecular pathogenesis of PTCs. To characterize the somatic alterations associated with PTCs, we performed whole-exome sequencing (WES) of PTCs from 23 Chinese patients. This study revealed somatic mutations in genes with relevant functions for tumorigenesis, such as BRAF, BCR, CREB3L2, DNMT1, IRS2, MSH6, and TP53. We also identified novel somatic gene alterations which may be potentially involved in PTC progression. Gene set enrichment analysis revealed that the cellular response to hormone stimulus, epigenetic modifications, such as protein/histone methylation and protein alkylation, as well as MAPK, PI3K-AKT, and FoxO/mTOR signaling pathways, were significantly altered in the PTCs studied here. Moreover, Protein-Protein Interaction (PPI) network analysis of our mutated gene selection highlighted EP300, KRAS, PTEN, and TP53 as major core genes. The correlation between gene mutations and clinicopathologic features of the PTCs defined by conventional ultrasonography (US) and contrast-enhanced ultrasonography (CEUS) were assessed. These analyses established significant associations between subgroups of mutations and respectively taller-than-wide, calcified, and peak time iso- or hypo-enhanced and metastatic PTCs. In conclusion, our study supplements the genomic landscape of PTCs and identifies new actionable target candidates and clinicopathology-associated mutations. Extension of this study to larger cohorts will help define comprehensive genomic aberrations in PTCs and validate target candidates. These new targets may open methods of individualized treatments adapted to the clinicopathologic specifics of the patients.

## Introduction

Papillary thyroid carcinomas (PTCs) represent the most common histological subtype of thyroid carcinoma (THCA), accounting for 75 to 85% of cases, and the incidence of which has increased rapidly in past decades 1, 2. PTCs are usually curable, with a 5-year survival rate of over 95% 3. However, lymph node metastasis or postoperative recurrence still occurs in 30% of cases 4. Until now, the mechanisms underlying the tumorigenesis and development of PTCs have remained largely unknown. Reliable markers to predict or evaluate tumor progression and prognosis are still required.

Recently, progress was made toward an understanding of the molecular mechanisms leading to THCA pathologies. In particular, advances in sequencing technology provide promising methods to identify somatic mutations and altered signaling pathways during PTC progression 2, 5. This technology already uncovered potential tumor-initiating gene mutations among which are: BRAFV600E-like and RAS-like cohorts related to the MAPK signaling pathway, mutations affecting AKT1, PIK3CA, and PTEN all associated with the PI3K signaling pathway, and alterations of CHEK2, EIF1AX, and PPMID. The Cancer Genome Atlas (TCGA) project also identified the TERT promoter mutation, which accounts for approximately 1% of PTCs and is associated with a high risk of recurrence 2. Combined analyses of genomic variants, gene expression, and methylations demonstrated that different driver groups lead to different pathologies with distinct signaling and differentiation characteristics. This information may be used to define predictors in PTC pathologies.

Although next-generation sequencing (NGS) has yielded many promising results in PTCs, the majority of the studies uncovered only well-recognized multiple PTC-related somatic mutations and signal pathways 6-9. To our knowledge, NGS was rarely applied to compare the genetic backgrounds and clinical characteristics of PTCs in attempts to correlate genetic changes and clinicopathological features. Ultrasonography (US) is considered the most important imaging tool for thyroid disease characterization and diagnosis. Therefore, we propose that combining US and NGS can provide better information for disease management. To this aim, we carried out whole-exome sequencing (WES) of the PTCs of 23 patients and investigated their genetic profiles. We characterized the genetic mutations and corresponding pathways altered during the progression of PTCs, and novel driver gene candidates susceptible to underlying carcinogenesis development. Further, correlations between different sets of mutations and clinicopathological features of the PTCs were assessed to explore novel biological markers for prognosis, individual diagnosis, and treatment of PTCs.

## Materials and methods

### PTCs patients and tissues sampling

PTCs samples were collected from 23 patients who underwent thyroidectomy in the Affiliated Hospital of Yangzhou University, China, from May to November 2019. The tissues were immediately frozen in liquid nitrogen and stored at -80°C for later analyses. All of the patients were diagnosed with PTC pathologies according to the World Health Organization classification, and the tissues were reviewed by two pathologists. None of the patients had received radioactive iodine treatment or other therapies before sample collection. The baseline characteristics of the patients were collected from their clinical records and summarized in **Table 1**. This study received ethical approval from the Ethics Committee of the Affiliated Hospital of Yangzhou University, China. All of the patients or their families signed informed consent before the study.

### US examination and imaging analysis of the PTCs

We performed the conventional US and contrast-enhanced ultrasonography (CEUS) examination for each PTC nodule using a MyLab Twice US system (Esaote SpA, Genoa, Italy) equipped with a linear volumetric array transducer (BL433). To limit operator-related variability, all of the examinations and image analyses were performed by one sonographer with more than 10 years of experience in US diagnosis and more than 5 years of experience in performing CEUS of thyroid nodules. For CEUS, the transducer was set on the long axis, avoiding blood vessels and choosing the largest section of the nodule. Using a 20-gauge peripheral intravenous cannula, a bolus consisting of a 2-ml dose of contrast agent (Sonovue, Bracco, Italy), followed by 5 ml of 0.9% saline flush were injected. Each contrast imaging acquisition lasted for at least 1 min after bolus injection; the images were stored digitally as raw data on the internal hard disk and then exported to an external workstation for subsequent analysis.

Using the conventional US and according to the American College of Radiology (ACR) Thyroid Imaging Reporting and Data System (TI-RADS)^10^, for each PTC nodule, we gathered the following information (see **Table 1**): maximum diameter, shape (taller-than-wide or wider-than-tall), margin (smooth, ill-defined, lobulated, or irregular and extra-thyroidal extension), echogenicity (anechoic, hyperechoic, or isoechoic, hypoechoic, and very hypoechoic), and calcification (none, macrocalcifications, and microcalcifications). CEUS enhancement type (hypo-, hyper-, or iso-enhancement) of each PTC nodule was also determined. Cervical lymph node metastasis status (absent or present) was determined according to the postoperative pathological results.

### Genomic DNA extraction and sequencing

Genomic DNA was extracted from the 23 PTCs samples with DNeasy Blood & Tissue Kit (Qiagen, Hilden, Germany) following the manufacturer's instructions. The quantity and quality of the isolated DNA were determined by Qubit dsDNA HS Assay Kit (Life Technologies, Darmstadt, Germany) on the Qubit 2.0 Fluorometer, and by 1% agarose gel electrophoresis analysis, respectively. Target genes were amplified using NEXTflex™ PCR Master Mix according to the manufacturer's instructions (Cat5144-02, Bioo Scientific, USA). The genomic DNA libraries were prepared by the IlluminaTruSeq DNA Sample Prep Guide (Illumina, San Diego, CA, USA). After amplification and purification, the quality of the libraries was assessed by an Agilent 2100 Bioanalyzer (Agilent Technologies, San Jose, CA, USA). Exomes were captured by an Agilent SureSelect paired-end version 2.0 human exome kit (Agilent, Santa Clara, CA) following the manufacturer's instructions. An Illumina HiSeq3000 Genome Analyzer platform (Illumina, San Diego, CA) was used for whole-exome sequencing.

Another 64 PTC patients' tissues were collected and Sanger sequencing was used to support the somatic mutation profiling's authenticity and verify the frequency of mutations in genes, BRAF (rs113488022-F: ACCTCATCCTAACACATTTCAAGC; rs113488022-R: GTAACTCAGCAGCATCTCAGGG), TP53 (rs1042522-F: TGGTAAGGACAAGGGTTGGG; rs1042522-R: GGGAAGGGACAGAAGATGACA), MSH3 (rs144607594-F: TTGACAGAAGAAAGAAGAGACCATT; rs144607594-R: AACCTCCTATACACATCACCATCAC) and MSH6 (rs55740729-F: AAAGGGGAAGGGATGATGC; rs55740729-R: TGGTGACAGTGGGTATAAAACAGC) were selected.

### Somatic variations analysis

The raw sequence data were aligned to the GRCh37 human reference genome using BWA 0.7.17-r1188 (http://bio-bwa.sourceforge.net/) 11. PCR duplicates were marked using the Mark Duplicates program in the Picard-tools-1.115 toolset (https://github.com/broadinstitute/picard). The Depth of Coverage functionality of the Genome Analysis Tool Kit (GATK-v4.1.4.1) (https://software.broadinstitute.org/gatk/) 12 was used to identify INDELs (insertions and deletions). Alignment coverage was calculated using bedtools (http://bedtools.readthedocs.io/en/latest/) 13. Mutect2 (http://samtools.sourceforge.net/) 14 was used to call the single nucleotide variants (SNVs) and small INDELs. All of the SNVs were functionally annotated using the snpEff program (http://snpeff.sourceforge.net/) 15. The isolated SNVs were further purged of known variants annotated in the 1000 Genomes Project (global freq > 0.01) and the Exome Aggregation Consortium (ExAC) databases (global freq > 0.01). The exclusion of synonymous and known polymorphisms led to the discovery of novel missense and nonsense SNVs and splice-site variants. Tumor-related genes were screened using Oncology Knowledge Base (OncoKB) 16 and Kyoto Encyclopedia of Genes and Genomes (KEGG) 17 tumor online databases.

### Mutational gene interaction network analysis

The curated SNVs were analyzed impartially for their impact predictions on structural, functional, and evolutionary properties of their corresponding protein products using validated bioinformatics databases. Pathway and network enrichment analyses were performed by using Gene Ontology (GO) and KEGG databases through Metascape (http://metascape.org/gp/), with a false discovery rate of < 0.5 18. Protein-Protein Interaction Network (PPI network) analysis was performed with the Search Tool for the Retrieval of Interacting Genes/Proteins (STRING, https://string-db.org). The minimum required interaction score was set at medium confidence of (0.4). Mutually exclusive (ME) and co-occurring (CO) associations of the candidate genes were evaluated with the maftools of R-software. The significance threshold of the p-values was set at 5% and analyzed in R-software (version 2.15.2: http://www.cran.rproject.org/).

### Relationship analysis between somatic variations and PTCs clinical characteristics

Fisher's exact test was used to compare the clustering differences among groups of patients' clinical data collected according to the ACR TI-RADS. A *p-value* < 0.05 was considered to be significantly different.

### Statement

We confirmed that all methods were performed following the relevant guidelines and regulations.

## Results

### Baseline characteristics of the studied population

23 eligible patients were recruited for WES analysis in the study. 14 were females, and the average age was 50.43 ± 7.69 years; 10 patients' PTCs were classified as taller-than-wide, and only 1 patient presented with a well-defined margin PTC; microcalcifications were observed in 9 patients' nodules; CEUS detected iso- or hypo-enhancements at the peak time for 8 patients. Also, 7 patients presented lymph node metastasis. The clinical characteristics of the patients are summarized in **Table 1**.

### The landscape of mutations in patients' PTCs

**Figure 1:**
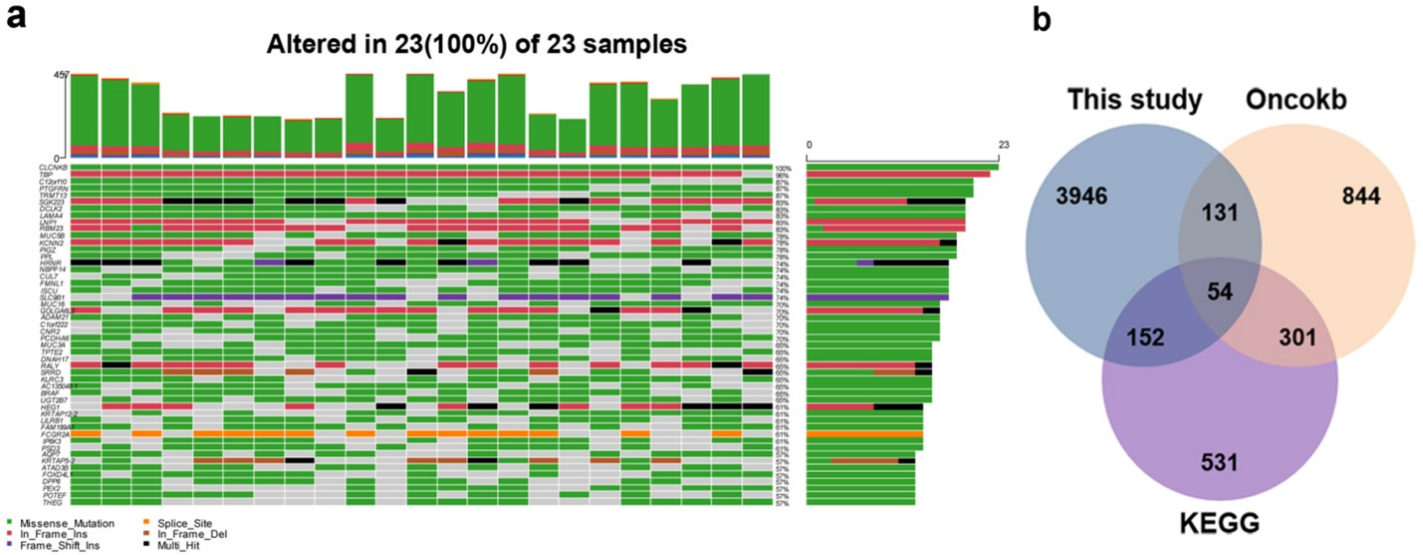
** Landscape of mutations in patients' PTCs. a** Mutational Landscape of the top 100 genes affected in the 23 PTCs. **b** Venn diagram indicated 54 genes of crucial cancer-related functions after comparative analyses with KEGG and OncoKB databases.

To detect SNV mutations, WES was performed on the DNA extracted from the 23 PTC samples. All of the SNVs were then filtered according to the following exclusion criteria: 1) synonymous mutations; 2) variants with a minor allele frequency (MAF) > 1% according to 1000 Genomes and ExAc databases; and 3) introns, intergenic, and Untranslated Regions (UTR) sites. The 23 samples involved in the study displayed a median of 953 exonic mutations (range, 388-1076) per sample identified by NGS analysis, affecting a total of 3350 genes (**Supplemental Table 1**). The mutational landscape of the top 50 genes is shown in **Fig. 1a**. Missenses (41.3%) and splice sites (12.3%) were the two prominent types of alteration. Comparative analyses with KEGG and OncoKB databases excluded genes of non-relevant functions regarding cancer biology and retained 54 genes of crucial cancer-related functions (**Fig. 1b, Supplemental Table 2**).

### SNVs in the mutant genes

**Figure 2:**
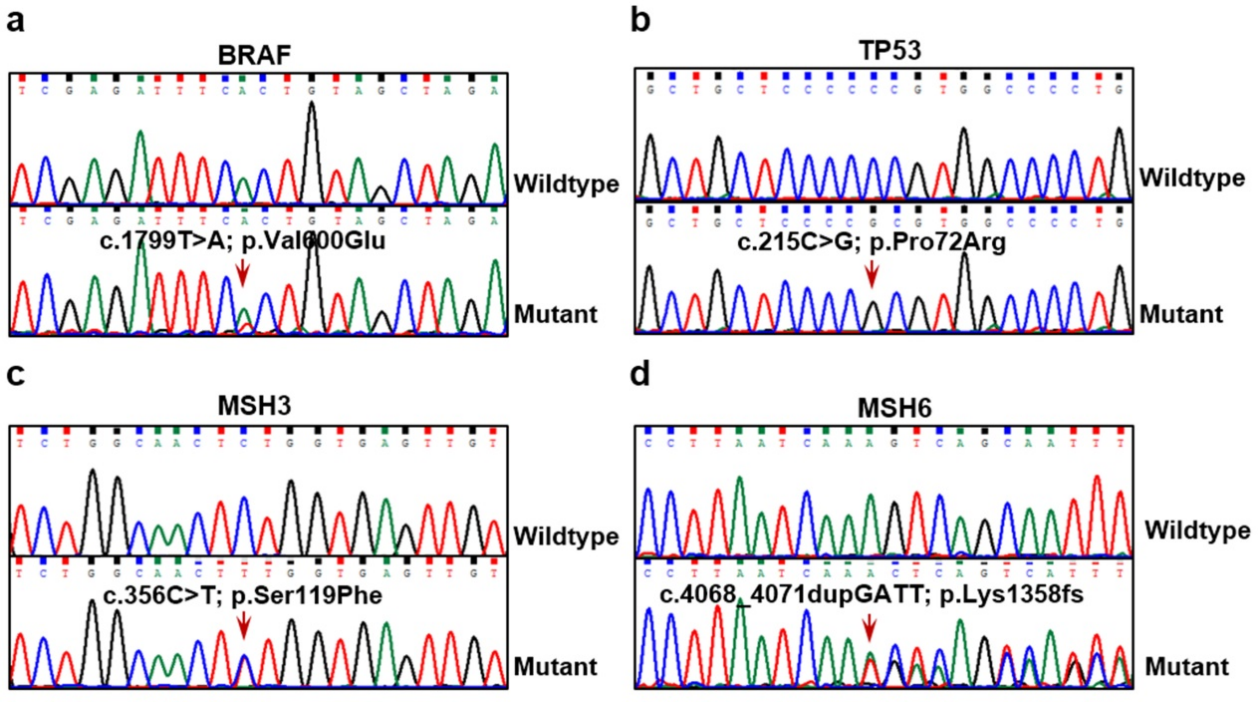
** BRAF, TP53, MSH3, and MSH6 mutations identified in PTC tissue samples.** Mutation sites of BRAF c.1799T>A (**a**), TP53 c.215C>G (**b**), MSH3 c.356C>T (**c**) and MSH6 c.4068_4071dupGATT (**d**) detected in PTC tumor tissues by Sanger sequencing. Lower panels show sequencing traces of mutations identified in PTC tissue samples, and top panels show sequencing traces for normal examples.

We identified 69 SNVs sites across the 54 cancer-related genes discovered during the analysis of the 23 PTC samples (**Supplemental Table 3**). 28 of these SNVs were in exons (**Table 2**). The 10 most frequently altered genes were: BRAF (65.22%), BCR (56.52%), CREB3L2 (39.13%), IRS2 (21.74%), DNMT1 (17.39%), MSH6 (17.39%), TP53 (17.39%), CREB3L1 (13.04%), MSH3 (13.04%) and SMARCA2 (13.04%). Moreover, the frequency of BRAF mutations reflected closely that reported in the Catalogue of Somatic Mutations in Cancer (COSMIC) database (**Supplemental Fig. 1**). Another 64 PTC patients' tissues were collected and several important mutated genes, such as BRAF, TP53, MSH3, and MSH6, were selected to verify their mutation frequency and support the somatic mutation profiling's authenticity (**Fig. 2**). In our verification cohort, the mutation frequencies of BRAF, TP53, MSH3, and MSH6 were 45.31% (29/64), 34.38% (22/64), 7.81% (5/64), and 12.50% (8/64), respectively, which did not fully consistent with our WES results. This is probably the reason for the different samples and other factors, but it was within the range of mutation frequency reported in the previous studies 6, 19. We also found that in the 23 PTCs samples, BCR, CREB3L1, and MSH6 underwent frameshift changes, a type of variation likely to affect the structure and function of these 3 gene products in the PTCs. These altered expressions may be involved in the occurrence of the PTCs.

### Identification of novel mutation sites in PTCs

In addition to the confirmed SNVs mentioned above, we also found mutations in plenty of novel genes potentially associated with the genesis of PTCs (**Supplemental Table 4**). Among the 54 cancer-related genes, DGKG, MPND, NPIPB5, PRR21, SGSM1, and TRIM67 displayed frameshift mutations. The mutation ratios of these 6 genes ranged from 8.70% to 21.74% (**Table 3**). Further, the frameshifts found in MPND, PRR21, and TRIM67 were in the first 80 amino acids of their coding regions. This alteration may cause the complete loss of function of the 3 genes and have a significant connection with PTC genesis.

### GO and KEGG pathway analyses of cancer-related mutant genes in PTCs

**Figure 3:**
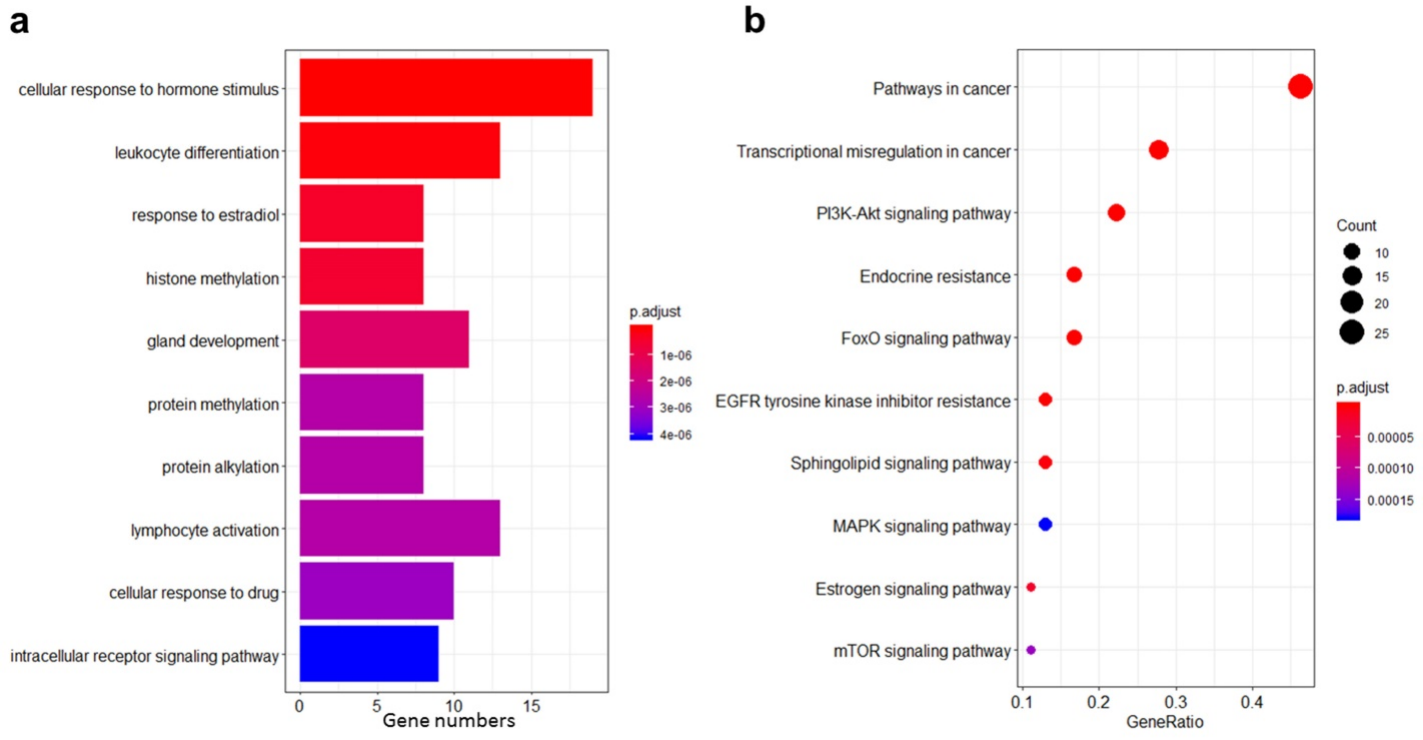
** GO and KEGG pathway analyses of cancer-related mutant genes in PTCs. a** Ten cancer-related GO biological processes result from the gene set enrichment analysis performed on the 54 cancer-related mutant genes in the 23 PTCs.** b** Ten cancer-related KEGG pathways enriched by the 54 cancer-related mutant genes in the 23 PTCs.

To better understand the potential mechanisms underlying PTC genesis, we performed GO and KEGG pathway analyses on the selected 54 cancer-related mutant genes. The most significant result from the gene set enrichment analysis performed in GO Biological Processes was “cellular response to hormone stimulus” (**Fig. 3a**). Another term related to hormone response was also found, i.e., “estradiol response.” Besides, this analysis highlighted several significantly differential terms linked to protein modifications, such as “histone methylation,” “protein methylation,” and “protein alkylation.” These latter features are affected in most types of cancer. Conversely, the finding that pathways related to “gland development” were significantly different in this study is worth emphasizing and may represent an important specificity of THCA.

KEGG pathway analysis showed that “endocrine resistance” and “estrogen signaling” were the most significant pathways besides common cancer pathways (**Fig. 3b**). These results are in keeping with the GO gene set enrichment analysis and suggest that hormone responses are of particular importance in PTCs. Other pathways commonly associated with cancer, such as EGFR, MAPK, mTOR, and PI3K-Akt, were also significantly enriched.

### PPI Network and co-occurring of the mutated genes

**Figure 4:**
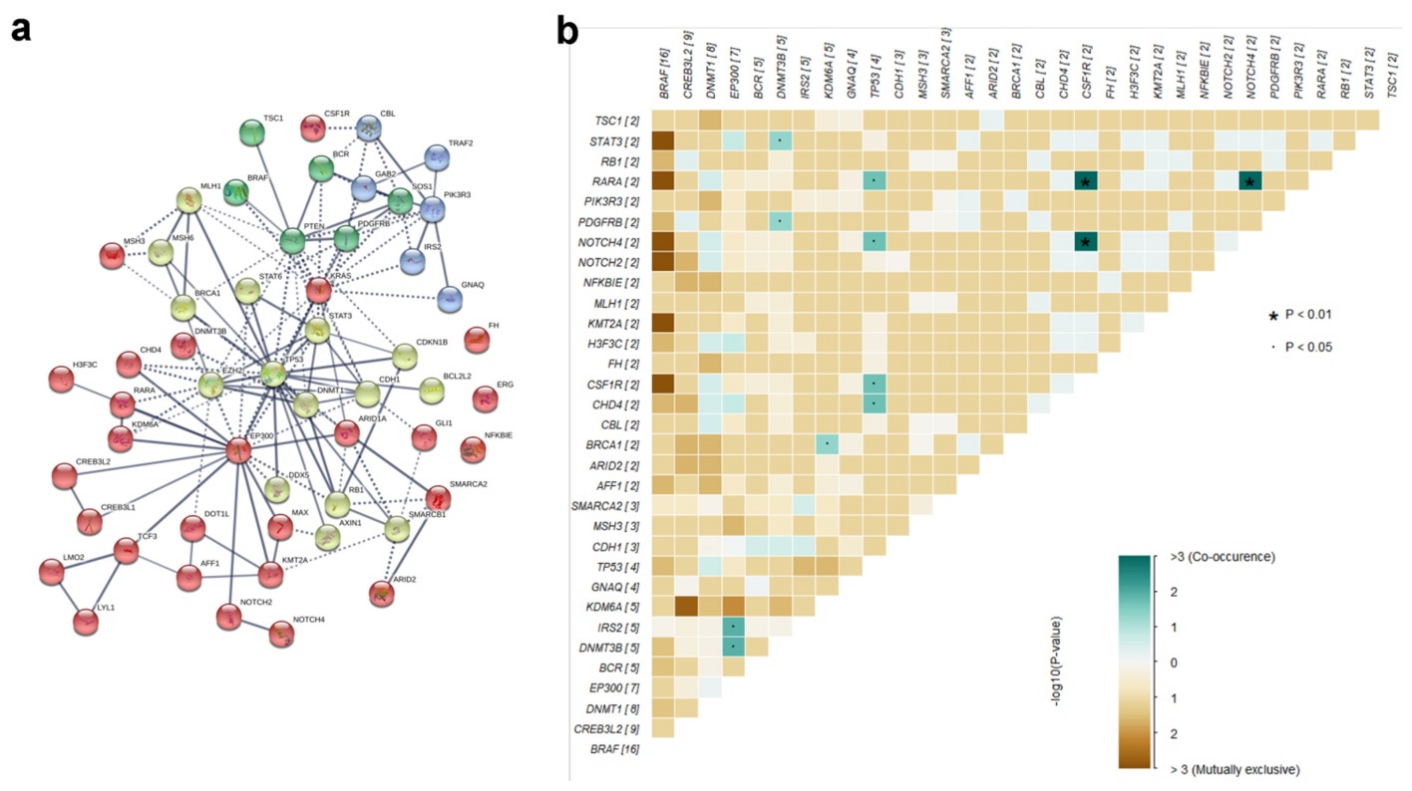
** PPI Network and co-occurring of the mutated genes. a** STRING analysis of the mutant genes, and showed that EP300, KRAS, PTEN, and TP53 were the major core genes of the network.** b** Co-occurring analysis of the mutant genes, TP53 and EP300 co-occurred with several other gene mutations with significant scores.

To further investigate the genetic interactions in PTCs, we analyzed our set of the 54 mutant genes for PPI Networks and co-occurring genes. STRING analysis pointed out EP300, KRAS, PTEN, and TP53 as major core genes of the network, each connecting more than 10 other genes **(Fig. 4a)**. By the PPI network, the co-occurring analysis indicated that mutations in TP53 and EP300 co-occur with several other gene mutations with significant scores (**Fig. 4b, Supplemental Table 5**). Mutations in TP53, a well-known tumor suppressor gene, co-occur with mutations in CHD4, CSF1R, NOTCH4, and RARA (all with *p*=0.024). Mutations in EP300 co-occur with mutations in DNMT3B (*p*=0.017), IRS2 (*p*=0.017), CHD4 (*p*=0.083), and H3F3C (*p*=0.083). Co-occurring CHD4 mutations with mutations in both EP300 and TP53, concomitantly with connections to these three genes in the PPI network, suggest that TP53, EP300, and CHD4 are key factors in PTC genesis.

### Differential mutant gene representations in cluster analysis

To perform cluster analyses of the mutated genes (all mutant genes that intersect with KEGG or Oncokb databases, a total of 337) found in the 23 PTC samples, we defined 2-3 groups within each class of clinical indicators, i.e., age, gender, maximum diameter, shape, margin, echogenicity, calcification, CEUS enhancement type, and cervical lymph node metastasis. Using Fisher's exact test, we found several statistically significant associations between gene mutations and clinical criteria, as reported in **Table 4**. Mutations in TNN (rs386636935) and CLTCL1 (rs3747060) were differentially represented across gender groups; mutations in BRAF (rs113488022) and CDH11 (rs1520238) were significantly different between PTCs of different shapes; the group of mutations among others LDHAL6A (rs2279902), PRKDC (rs11411516, rs397814002), CREB3L2 (rs3217268, rs66593747), DGKZ (rs185718937), and CYP2C18 (rs1126545) were significantly different across the calcification groups; the group of mutations among others GSTT2 (rs35389228, COSM4002112), PRKAR1A (rs8082254), HDAC5 (rs398030911), KLF2 (rs3745319), and IL15RA (rs41294171, COSM3769142, COSM3769143) were statistically different across the different CEUS-enhancement types, and finally, mutations of SMARCA2 (rs2296212), FZD8 (rs71003903), and NCOR2 (rs368678225) were significantly different according to the presence or absence of cervical lymph node metastases. These results indicate possible correlations between the gene mutation profiles and the clinical characteristics of PTCs, which may help provide candidate targets for better diagnosis and tailored therapies in PTC management.

## Discussion

PTC is a complex disease of which the pathogenesis is still unknown. The heterogeneity of its biological behavior may be attributed to diverse genetic backgrounds, molecular mutations, and environmental factors, such as iodine intake and radiation exposure 20-22. The development and application of sequencing technologies provide new ways to identify the multitude of mutated genes and changes in expression patterns during tumorigenesis and to investigate the molecular pathogenesis of PTCs. For example, a study from Yang *et al.* provided a list of novel candidate genes presenting somatic alterations of significant functional impact and may function as biomarkers in PTCs 6. Smallridge RC *et al.* compared BRAF-mutated and BRAF wild-type (BRAF-WT) tumors by RNA sequencing and identified 560 differentially expressed genes. The four most overexpressed genes in BRAF-WT tumors, i.e., IL1B; CCL19, CCL21, and CXCR4, belong to the immune function family and are correlated with lymphocyte infiltration 23. Leeman-Neill RJ *et al.* reported the ETV6-NTRK3 rearrangement, which was detected in 9 out of 62 (14.5%) post-Chernobyl PTCs and in 3 out of 151 (2%) sporadic PTCs. Moreover, the authors demonstrated that this rearrangement is significantly more common in tumors associated with Iodine131 exposure and has a borderline significant dose-response. These findings suggest that ETV6-NTRK3 rearrangement is a key mechanism in radiation-induced PTC carcinogenesis 24.

Herein, we applied the WES technology to investigate the somatic variations occurring during PTC tumorigenesis in the 23 Chinese PTC samples. To better identify the potential pathogenic sites in PTCs across all the mutants, we first intersected the thyroid oncogenes from the Oncokb and KEGG databases with our results, filtering out most of the genes that were unrelated or not closely related to THCA, thus effectively helping us to screen for the most likely pathogenic gene mutations in PTCs. And 54 crucial cancer-related genes were retained. Previous studies demonstrated the presence of a universal mutation in the BRAF gene, regarded as a reliable molecular marker associated with oncogenesis, invasion, and prognosis in PTCs. It was reported that the prevalence of BRAF mutations in PTCs is 30-80% 25, and Chinese patients with PTCs have a higher frequency of BRAF mutations 19, 26-29. In the present study, mutations in the BRAF gene were observed in 65% of the cases. Besides, many other genes are recurrently altered in PTCs, including KRAS, PTEN, TP53, and so on. Several important mutated genes, such as BRAF, TP53, MSH3, and MSH6, were selected to verify the mutation frequency, and found that the cohort verification results were not completely consistent with the results obtained by the WES. We believe that different samples, sampling conditions, operating procedures, and other factors may lead to the inconsistent results, but the mutation frequency is within the range reported previously 6, 19. Our verification cohort, at least to some extent, supported the authenticity of the somatic mutation profiling. It is noteworthy that the cohort validation results further confirmed the correlation between the high mutation rate of BRAF and pathogenicity, suggesting that BRAF mutation may be a valuable marker of PTCs and is significantly associated with the metastasis, prognosis, and increased cancer-related mortality of PTCs 30-32**.** Many other previously reported mutations were also found in our study, but with varying frequencies, suggesting heterogeneous genomic PTC landscapes among different races or ethnicities. In addition, the present study revealed 6 cancer-related genes, DGKG, MPND, NPIPB5, PRR21, SGSM1, and TRIM67, which showed novel frameshift mutations. We reviewed the articles about these 6 gene mutations and found that the relevant reports were rare, and up-to-date there is no report on thyroid tumors. Diacylglycerol kinases (DGKs) are important regulators of cell signaling and have been implicated in human malignancies. DGKG, which encodes DGKγ, its important role in cancer has been reported by many authors, Guo *et al*. 33 demonstrated that DGKγ plays a tumor suppressor role in HCC by negatively regulating GLUT1. Kai M *et al*. 34 found DGKG methylation was frequently observed in primary CRCs and was positively associated with KRAS and BRAF mutations, functional studies suggest that DGKG may play a tumor suppressor role in CRC. It is short of the report for mutations of DGKG, Ng.M C *et al*. 35 found that the ETV5/DGKGrs7647305T allele, which was protective for obesity in Europeans but at risk for T2D in their study, particularly after adjustment for BMI. MPND gene was cloned from retinoblastoma and encodes a 48.5 KD protein of the deubiquitinase family. MPND was reported to be dysregulated and associated with poor prognosis in gastric cancer. Whole-genome and transcriptome sequencing by Zhang *et al*. 36 revealed GPX4 and MPND in 19q13.3-13.4 region, is characterized as a novel fusion-gene, which causes up-regulation of mRNA expression of both genes in primary gastric cancer and may facilitate tumor growth and progression. The PRR21 gene encodes a putative proline-rich protein 21. Snezhkina, A V *et al*. 37 searched for genes with high mutation rate by using the whole-exome sequencing data of 52 Carotid paragangliomas (CPGLs) obtained earlier, 34 genes (including PRR21) potentially associated with the CPGL initiation and progression were revealed, and was first shown involvement in the development of paragangliomas/pheochromocytomas. In 2007, Yang *et al*. 38 reported a novel protein, SGSM1, which is especially expressed in the brain and appears to be associated with the small G protein-mediated neuronal signal transduction and vesicular transportation pathways. Zhang *et al*. 39 found silencing SGSM1 abrogated the inhibitory effect of SHISA3 on NPC cell migration and invasion. Tripartite motif-containing 67 (TRIM67), an E3 ubiquitin ligase, belongs to the TRIM protein family. It has been reported that TRIM67 plays a significant role in the progression of various tumors by activating different pathways 40-42. Although there are few reports on the mutations of these genes identified in our study, the above-published researches are sufficient to demonstrate that these genes have considerable biological functions in the pathological process. Our results provide a significant contribution to the genomic landscape of PTCs. Follow-up studies on the functions and mechanisms of these gene mutations in the occurrence and development of PTC may be of great value for clarifying its pathogenesis.

Using available databases and bioinformatics, we further determined crucial biological processes and signaling pathways likely to be affected by the somatic variations observed in the 23 PTCs of this study. Studies have shown that PTC is a hormone-dependent tumor, and hormones can affect the growth activity of papillary carcinoma. For example, the serum TSH level is correlated with the incidence and severity of PTC patients 43, 44**,** and estrogens can influence PTC cell growth 45, 46. In this study, gene set enrichment analysis performed on GO Biological Processes revealed a group of genes belonging to the “cellular response to hormone stimulus”, which is one of the main ways to be affected in PTCs. It is suggested that mutant genes may affect the hormone level and then stimulate thyroid cells through the process of “cellular response to hormone stimulus” to promote the occurrence and development of PTCs. Of particular interest, epigenetic modifications, such as protein methylation, histone methylation, and protein alkylation, were enriched. Alterations in methylation patterns have been demonstrated in a variety of malignancies, including PTCs. For example, the tumor suppressor PTEN was reported to be epigenetically hypermethylated in PTCs, a modification that affects the expression of other canonical pathways, such as that of the PI3Ks 47, 48. Likewise, the histone methyltransferase KMT5A is overexpressed in PTCs, and associates with advanced pathological stages, such as extrathyroidal extension and lymph node metastasis, acting as a tumor gene that modulates oncogenesis and lipid metabolism of PTCs 49. Usual signaling pathways, such as MAPK, PI3K-AKT, and FoxO/mTOR, were significantly enriched in our study. It is plausible that variations in BRAF, the gene most frequently mutated in PTCs, activate the MAPK signaling pathway, thereby promoting the aggressive progression of PTCs 50-52, and activation of MAPK is indeed considered to be a primary mechanism for PTC initiation. Abdul K. Siraj* et al*. 53 observed mutations in MAPK-signaling-pathway genes play a major role in PTC pathogenesis, the MAPK-pathway gene mutations are seen at a relatively high frequency of 66.6%; Hou P *et al*. 54 found the progression of PTC to anaplastic thyroid carcinoma (ATC) may be facilitated by the coexistence of PI3K/Akt pathway-related genetic alterations and BRAF mutation. The findings of this study are consistent with these previous reports, at least reflecting the reliability of our data. We also identified TP53, EP300, KRAS, and PTEN were the major core genes of the interaction network in our study, a large number of studies have confirmed the importance of these key genes in the pathogenesis of tumors, and PTC is no exception 6, 55-61. Mutations in TP53 in PTCs have been reported in many studies 62-64, and they are significantly associated with the monitored tumor characteristics. Moreover, the TP53 mutation is thought to play a critical role in the transformation of differentiated thyroid cancer into anaplastic thyroid cancer 65, 66. In this study, although our sample size is relatively small, we have obtained many valuable mutant genes and enriched some significant biological functions and signal pathways. However, it is necessary to conduct validation analysis of the findings based on expanding the samples and combining them with subsequent experiments, which will be the focus of our future studies.

Currently, TI-RADS is used to evaluate the benign and malignant degrees of thyroid nodules by conventional ultrasound 67, 68. Characteristic ultrasonic manifestations, such as margin roughness, irregular shape, taller-than-wide shape, low echo or very low echo inside the nodules, and microcalcification are important indicators for the diagnosis of PTCs. It has been reported that a taller-than-wide shape is an insensitive but highly specific indicator of malignancy 69, 70. In the solid components of thyroid nodules, microcalcifications may correspond to the psammomatous calcifications associated with PTCs and are therefore considered a highly suspicious malignancy risk 71, 72. CEUS can clearly detect microvessel blood flow in tumors, and it can accurately evaluate the sequence and intensity of tumor perfusion and vascularity 73, 74. The CEUS patterns of PTCs are not only relative to the size of PTC but also to the possibility of cervical lymph node metastasis after thyroidectomy. Iso- or hypo-enhancements at peak time seem to predict cervical lymph node metastasis prognoses in PTC patients 72. Also, cervical lymph node metastasis increases the risk of recurrence of PTCs and is associated with PTC-related deaths 75. In the study of Li *et al.* the comparative analysis of conventional US and CEUS diagnosis of PTCs showed that CEUS features, i.e., slow enhancement, late enhancement, and low enhancement, were highly correlated with PTC diagnosis, and that combined diagnosis with conventional US and CEUS is more sensitive and specific 76. Shi *et al.* 22 reported that taller-than-wide shape, microlobulated margin, capsule contact, or involvement on the US as well as age > 50 years, and multifocality were independent predictors of TERT promoter mutations in PTCs. Our results identified groups of mutated genes differentially represented in PTCs displaying taller-than-wide shape, microcalcifications, Iso- or hypo-enhancements at peak time, and cervical lymph node metastasis. These mutated genes may be significantly correlated with suspicious US features and clinicopathologic characteristics, but also tumor metastasis in PTC patients. The new genetic signatures may further contribute to the decision-making of genetic testing and FNA cytological examination, as well as patient management and follow-up.

**Figure 5:**
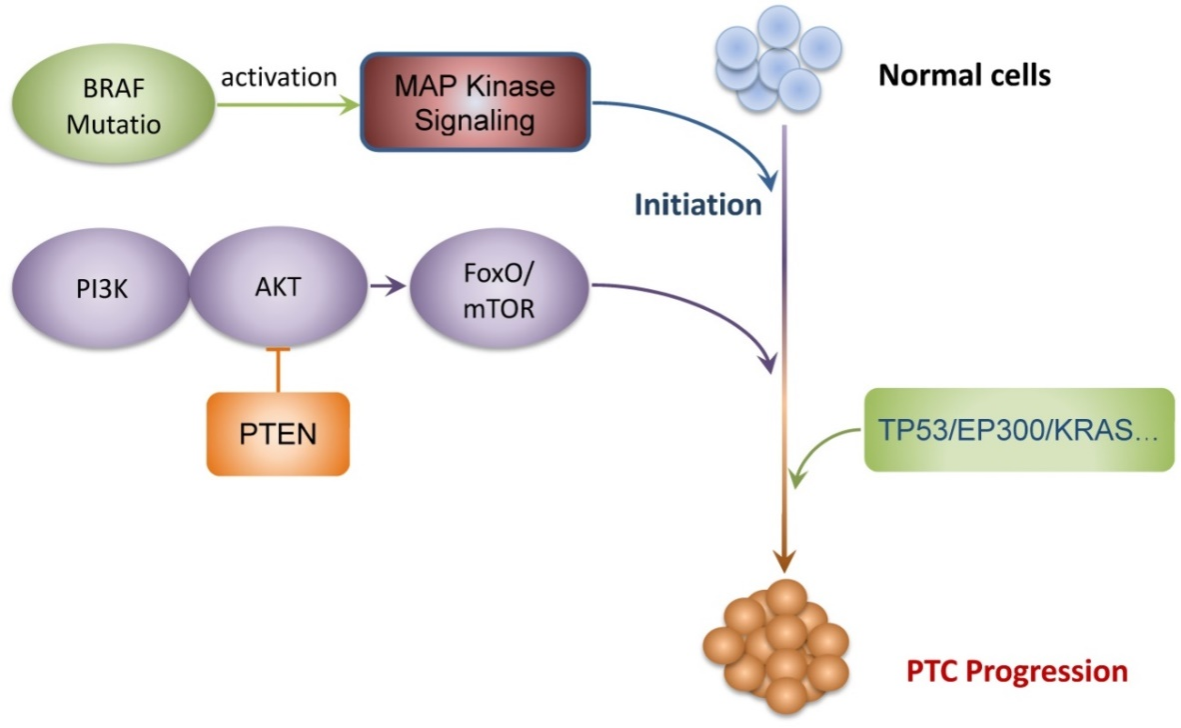
Schematic model of the speculative pathogenesis of PTC.

In conclusion, WES seems to be a promising approach to investigate the mutational landscape during tumorigenesis of PTCs and detected BRAF mutations that are universally found in PTCs. The gene set enrichment analyses performed on our selection of mutated genes suggest that PI3K-AKT-FOXO/mTOR and MAPK signaling pathways are implicated in the development and progression of PTCs. The interaction networks and co-occurring among the mutated genes identified that TP53, EP300, KRAS, and PTEN were the major core genes, suggesting the potential therapeutic targets for PTCs. Finally, significant correlations between the gene mutation profiles and the clinical characteristics of PTCs were established. In general, our study supplements the genomic landscape of PTCs and identifies new actionable target candidates and clinicopathology-associated mutations. We summarized a schematic model of the speculative pathogenesis and progression of PTCs, as shown in **Fig. 5.** These comprehensive genomic aberrations of PTCs and potentially actionable targets needed to be validated by large cohort studies, which may serve to be used in the development of individualized treatments in PTC patients.

## Supplementary Material

Supplementary figure S1.

Supplementary tables.

## Figures and Tables

**Table 1:** Baseline characteristics of 23 PTC patients in the study.

Subjects	Age	Gender	Maximum diameter (mm)	Conventional ultrasonic characteristics				Contrast-enhanced ultrasonography characteristics	Cervical lymph node metastasis^f^
				Shape^a^	Margin^b^	Echogenicity^c^	Calcification^d^	Enhancement type^e^	
W587	41	Male	12	1	1	1	0	1	0
L181	50	Female	13	0	1	1	0	0	0
G348	54	Male	9	1	1	1	0	0	1
X278	50	Male	20	0	0	1	1	1	0
J553	41	Female	7	1	1	1	0	0	0
X214	57	Female	8	1	1	1	1	0	0
Y252	57	Female	10	0	1	1	1	0	0
L245	23	Male	17	1	1	1	2	1	1
P148	54	Female	12	0	1	1	2	1	0
L579	23	Male	32	0	1	1	1	1	0
Q491	37	Female	15	0	1	1	2	1	1
W520	43	Male	8	1	1	1	0	0	0
P078	54	Male	14	0	1	1	2	0	1
B457	55	Male	33	0	1	1	0	1	1
J362	45	Female	11	1	1	1	1	0	0
Y257	50	Female	11	1	1	1	1	0	0
S312	38	Male	16	0	1	1	1	0	1
Z499	46	Female	9	0	1	1	0	0	0
L574	55	Female	18	0	1	1	1	1	0
F586	44	Female	13	1	1	1	1	0	0
B290	54	Female	11	0	1	1	2	0	0
Z543	67	Female	23	0	1	1	2	0	1
S318	49	Female	7	1	1	1	2	0	0

**Note: a** 0-Wider than tall; 1-Taller than wide. **b** 0-Well defined; 1-Poorly defined. **c** 0-Markedly hypoechoic; 1-Hypoechoic. **d** 0-Absent; 1-Micro; 2-Massive. **e** 0-Hypoenhancement; 1-Hyperenhancement or isoenhancement. **f** 0-Absent; 1-Present.

**Table 2:** 28 SNVs sites (count >1) in 54 cancer-relate genes' exons of 23 PTCs samples.

Gene	Chrom	Transcript	HGVS.c	HGVS.p	Ratio	Count
BRAF	chr7	ENST00000288602.6	c.1799T>A	p.Val600Glu	65.22%	15
CREB3L2	chr7	ENST00000330387.6	c.299_301delCCA	p.Thr100del	39.13%	9
BCR	chr22	ENST00000305877.8	c.3275_3278dupCCGG	p.Val1094fs	34.78%	8
BCR	chr22	ENST00000305877.8	c.3316G>A	p.Asp1106Asn	21.74%	5
IRS2	chr13	ENST00000375856.3	c.3170G>A	p.Gly1057Asp	21.74%	5
DNMT1	chr19	ENST00000359526.4	c.358G>C	p.Val120Leu	17.39%	4
MSH6	chr2	ENST00000234420.5	c.4068_4071dupGATT	p.Lys1358fs	17.39%	4
TP53	chr17	ENST00000269305.4	c.215C>G	p.Pro72Arg	17.39%	4
CREB3L1	chr11	ENST00000288400.3	c.1525dupG	p.Asp509fs	13.04%	3
MSH3	chr5	ENST00000265081.6	c.356C>T	p.Ser119Phe	13.04%	3
SMARCA2	chr9	ENST00000349721.2	c.4638C>G	p.Asp1546Glu	13.04%	3
AFF1	chr4	ENST00000395146.4	c.646C>G	p.Pro216Ala	8.70%	2
ARID2	chr12	ENST00000334344.6	c.1759A>G	p.Ser587Gly	8.70%	2
BRCA1	chr17	ENST00000471181.2	c.2566T>C	p.Tyr856His	8.70%	2
CBL	chr11	ENST00000264033.4	c.1858C>T	p.Leu620Phe	8.70%	2
CHD4	chr12	ENST00000309577.6	c.417G>T	p.Glu139Asp	8.70%	2
CSF1R	chr5	ENST00000286301.3	c.835G>A	p.Val279Met	8.70%	2
FH	chr1	ENST00000366560.3	c.77C>T	p.Pro26Leu	8.70%	2
H3F3C	chr12	ENST00000340398.3	c.116A>C	p.His39Pro	8.70%	2
KMT2A	chr11	ENST00000534358.1	c.9391G>A	p.Gly3131Ser	8.70%	2
MLH1	chr3	ENST00000231790.2	c.1151T>A	p.Val384Asp	8.70%	2
NFKBIE	chr6	ENST00000275015.5	c.275C>G	p.Ser92Trp	8.70%	2
NOTCH2	chr1	ENST00000256646.2	c.710G>A	p.Arg237Gln	8.70%	2
NOTCH4	chr6	ENST00000375023.3	c.45_47dupGCT	p.Leu16dup	8.70%	2
PDGFRB	chr5	ENST00000261799.4	c.1108C>T	p.Arg370Cys	8.70%	2
PIK3R3	chr1	ENST00000540385.1	c.902G>A	p.Arg301Gln	8.70%	2
STAT6	chr12	ENST00000300134.3	c.805G>A	p.Val269Ile	8.70%	2
TCF3	chr19	ENST00000395423.3	c.1322C>T	p.Ala441Val	8.70%	2

**Table 3:** Novel gene mutation sites in 23 PTCs samples.

Gene	Chrom	Transcript	HGVS.p	Variation.type	Ratio
DGKG	chr3	ENST00000265022.3	p.Gly712fs	frameshift_variant	21.74%
MPND	chr19	ENST00000596722.1	p.Glu20fs	frameshift_variant	17.39%
SGSM1	chr22	ENST00000400359.4	p.Glu886fs	frameshift_variant	13.04%
TRIM67	chr1	ENST00000366653.5	p.Ala61fs	frameshift_variant	8.70%
NPIPB5	chr16	ENST00000424340.1	p.Phe577fs	frameshift_variant	8.70%
PRR21	chr2	ENST00000408934.1	p.Met76fs	frameshift_variant	8.70%

**Table 4:** Differential sites of mutant genes in cluster analysis.

Grouping	Mutation sites	Gene	p_value	count_0	count_1	count_2
**Gender^a^**	rs386636935	TNN	0.0228	6/9	2/14	
	rs3747060	CLTCL1	0.0474	3/9	0/14	
**Shape^b^**	rs113488022	BRAF	0.0393	11/13	4/10	
	rs1520238	CDH11	0.0393	2/13	6/10	
**Calcification^c^**	rs2279902	LDHAL6A	0.0079	4/7	0/9	0/7
	rs11411516;rs397814002	PRKDC	0.0156	5/7	2/9	0/7
	rs3217268;rs66593747	CREB3L2	0.0159	6/7	2/9	1/7
	rs185718937	DGKZ	0.0196	3/7	9/9	6/7
	rs1126545;	CYP2C18	0.0196	4/7	0/9	1/7
	rs397773021;rs559752258;rs5898555	GNAQ	0.0196	4/7	0/9	1/7
	rs150689919;COSM5886696	TET1	0.0221	0/7	4/9	0/7
	rs199866252	SLC44A1	0.0221	0/7	4/9	0/7
	rs1520238	CDH11	0.0244	5/7	3/9	0/7
	rs150880809;rs71578334;rs765479702	NADK	0.0395	3/7	0/9	0/7
	rs8178232;440194;	PRKDC	0.0395	3/7	0/9	0/7
**Enhancement type^d^**	rs35389228;COSM4002112	GSTT2	0.0079	4/15	0/8	
	rs8082254;324800	PRKAR1A	0.0079	4/15	0/8	
	rs398030911;rs67111880	HDAC5	0.0272	5/15	7/8	
	rs3745319	KLF2	0.0316	3/15	0/8	
	rs41294171;COSM3769142;COSM3769143	IL15RA	0.0316	3/15	0/8	
	rs11243480	RAPGEF1	0.0329	1/15	4/8	
	rs1157181119	DGKZ	0.0393	4/15	6/8	
**Cervical lymph node metastasis^e^**	rs2296212	SMARCA2	0.0198	3/16	0/7	
	rs71003903	FZD8	0.0257	3/16	5/7	
	rs368678225	NCOR2	0.0450	2/16	4/7	

**Note:** a 0-Male; 1-Female. b 0-Wider than tall; 1-Taller than wide. c 0-Absent; 1-Micro; 2-Massive. d 0-Hypoenhancement; 1-Hyperenhancement or isoenhancement. e 0-Absent; 1-Present.
