# Supplementary figures and images for: Somatic Mutation Profiling of Papillary Thyroid Carcinomas by Whole-exome Sequencing and Its Relationship with Clinical Characteristics
Source: Int J Med Sci. 2021 Apr 26;18(12):2532–44. doi: 10.7150/ijms.50916 (PMC8176168; doi:10.7150/ijms.50916)

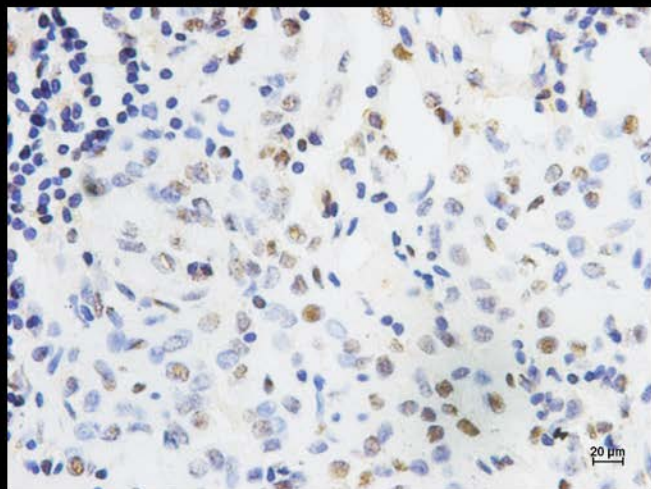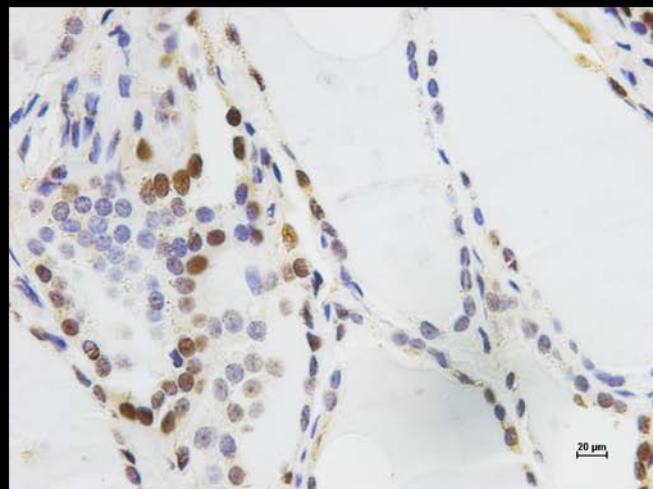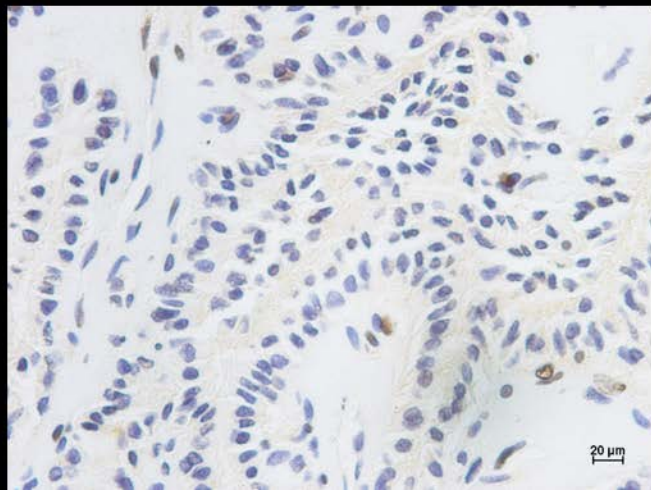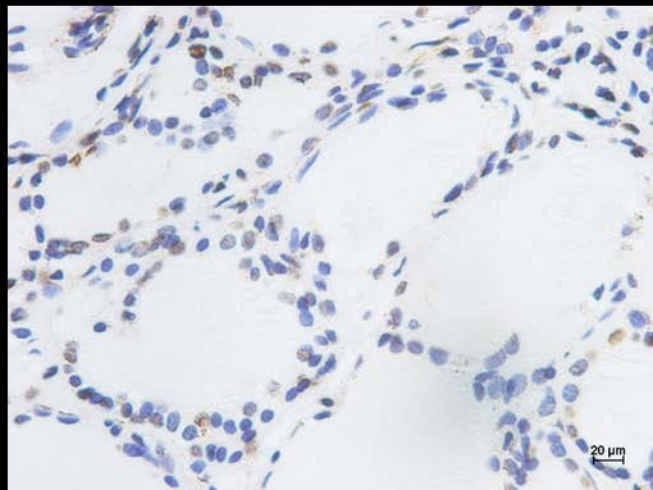

Supplement: Supplementary file 1 — Supplementary figure S1. [file ijmsv18p2532s1.pdf]
